# Effect of Casein Phosphopeptide-Amorphous Calcium Phosphate on the Microhardness and Fracture Resistance of Irradiated Permanent Human Teeth: An In Vitro Study

**DOI:** 10.7759/cureus.114168

**Published:** 2026-08-08

**Authors:** Divya Panday, Vatsal Chauhan, Nida Malik, Harshal S Chauhan

**Affiliations:** 1 Department of Dentistry, Rishiraj College of Dental Sciences and Research Centre, Bhopal, IND; 2 Department of Conservative Dentistry and Endodontics, K.M. Shah Dental College and Hospital, Vadodara, IND; 3 Department of Periodontics and Implantology, Kamla Nehru Hospital, Bhopal, IND; 4 Department of Information Systems, University of Maryland, Baltimore County, Baltimore, USA

**Keywords:** cancer, cpp-acp, dental enamel, fracture resistance, radiotherapy

## Abstract

Introduction

Radiotherapy, one of the most important modalities of treating head and neck cancer, causes xerostomia, mucositis, and structural and chemical changes in dental hard tissues. These factors favour the commencement of radiation-induced caries.

Aim

The study aimed to evaluate the changes in enamel microhardness and fracture resistance after irradiation and the effect of casein phosphopeptide-amorphous calcium phosphate (CPP-ACP) on the same.

Materials and methods

Fifty extracted human premolar teeth were debrided and cleansed using an ultrasonic scaler. In order to test for fracture resistance, 30 teeth were embedded in dental acrylic covering the entire root, leaving the crown exposed at the level of the cementoenamel junction (CEJ). For microhardness testing, 20 teeth were prepared by sectioning at the CEJ using a diamond disk with water. The crowns were sectioned longitudinally so as to keep the labial enamel, followed by mounting in acrylic resin. The samples were divided into the following groups: control, irradiation, and irradiation+CPP-ACP. The samples were subjected to irradiation for a total of 35 fractions equal to 70 Gy, with 2 Gy exposure per day applied five days per week, for seven weeks with X-rays from a linear accelerator (Elekta Infinity, Elekta AB, Stockholm, Sweden). In two groups, CPP-ACP (GC Tooth Mousse, GC Corporation, Tokyo, Japan) was applied onto the tooth surface using a microbrush and left for three minutes. The samples were tested for fracture resistance using a universal testing machine and surface microhardness using a microhardness tester. The data was analyzed using the Kruskal-Wallis test and Mann-Whitney U test.

Results

The mean microhardness of the control group was 323.50±7.76, the irradiation group was 269.60±9.41, and the irradiation and medicament group was 288.90±10.10. The mean fracture resistance of the control group was 1315.35±163.43, the irradiation group was 835.10±75.46, and the irradiation and medicament group was 1010.35±76.69. There was a significant difference between the groups for microhardness (a chi-squared value of 24.24 and a p-value of 0.001) and fracture resistance (a chi-squared value of 23.81 and a p-value of 0.001).

Conclusion

According to the findings of this study, we conclude that a radiation dose of 70 Gy alters microhardness and fracture resistance of enamel. The application of remineralizing agent CPP-ACP significantly improves the microhardness and fracture resistance of irradiated enamel. However, the application of CPP-ACP doesn't improve the microhardness and fracture resistance up to the level of a natural tooth.

## Introduction

Malignancies can be treated by a combination of surgery, chemotherapy, and radiotherapy. Radiotherapy has become an integral part of the treatment protocol for oral cancer [1]. But radiation has detrimental effects on healthy oral tissues, leading to xerostomia, mucositis, osteoradionecrosis, taste sensation loss, trismus, and structural and chemical changes in dental hard tissues. These factors favour the progression of radiation-induced caries [2].

Various tissues react at different rates to radiation exposure. Some tissues like salivary gland, taste buds, and mucosa show early changes, whereas bone shows late changes [3]. From the start to three months after the completion of radiation, hyposalivation causes an increase in the population of cariogenic bacteria, *Streptococcus mutans* and *Lactobacillus*, increasing the level of immunoglobulin A (IgA). These alterations further increase the chances of radiation-induced caries [4]. Owing to its aggressive nature, this type of caries is also called "rampant caries". It usually affects the cervical region of the labial surface of the tooth, an area which is generally resistant to caries due to the protective action of saliva [3,5]. Mucositis, xerostomia, and caries are responsible for dietary debilitation with difficult swallowing leading to a soft diet preference. Such patients suffer from nutritional deprivation, hence decreasing the patient's compliance towards cancer treatment [6]. With the advancement in medical science, the five-year survival rate of head and neck cancer is as high as 70% [7]. For the betterment of the quality of life of such patients, improvement in the management of side effects of radiotherapy is necessary.

Casein phosphopeptide-amorphous calcium phosphate (CPP-ACP) is a remineralizing agent. Casein phosphopeptide (CPP) is a peptide derived from milk which acts as a carrier for amorphous calcium phosphate (ACP). CPP stabilizes calcium phosphate resulting in a reservoir of calcium and phosphate in saliva [8]. The ions can be readily made available in the deficient area, aiding in the remineralization process. It is available in various forms, like toothpaste, mouthwash, tooth mousse, and chewing gums.

Various studies show that the microhardness of dentin is reduced due to radiation [3,9]. This could explain the involvement of cervical areas where break of the enamel-dentin junction would cause gap formation, enabling bacterial growth [3]. The aim of this study was to evaluate the changes in enamel microhardness and fracture resistance after irradiation and the effect of CPP-ACP on the same. The objectives of this study were, firstly, to evaluate the changes in enamel microhardness and fracture resistance after irradiation and, secondly, to evaluate the changes in microhardness and fracture resistance of irradiated teeth after CPP-ACP application. The null hypothesis tested was that CPP-ACP would not influence the microhardness and fracture resistance of irradiated human teeth.

## Materials and methods

Sample preparation

**Figure 1:**
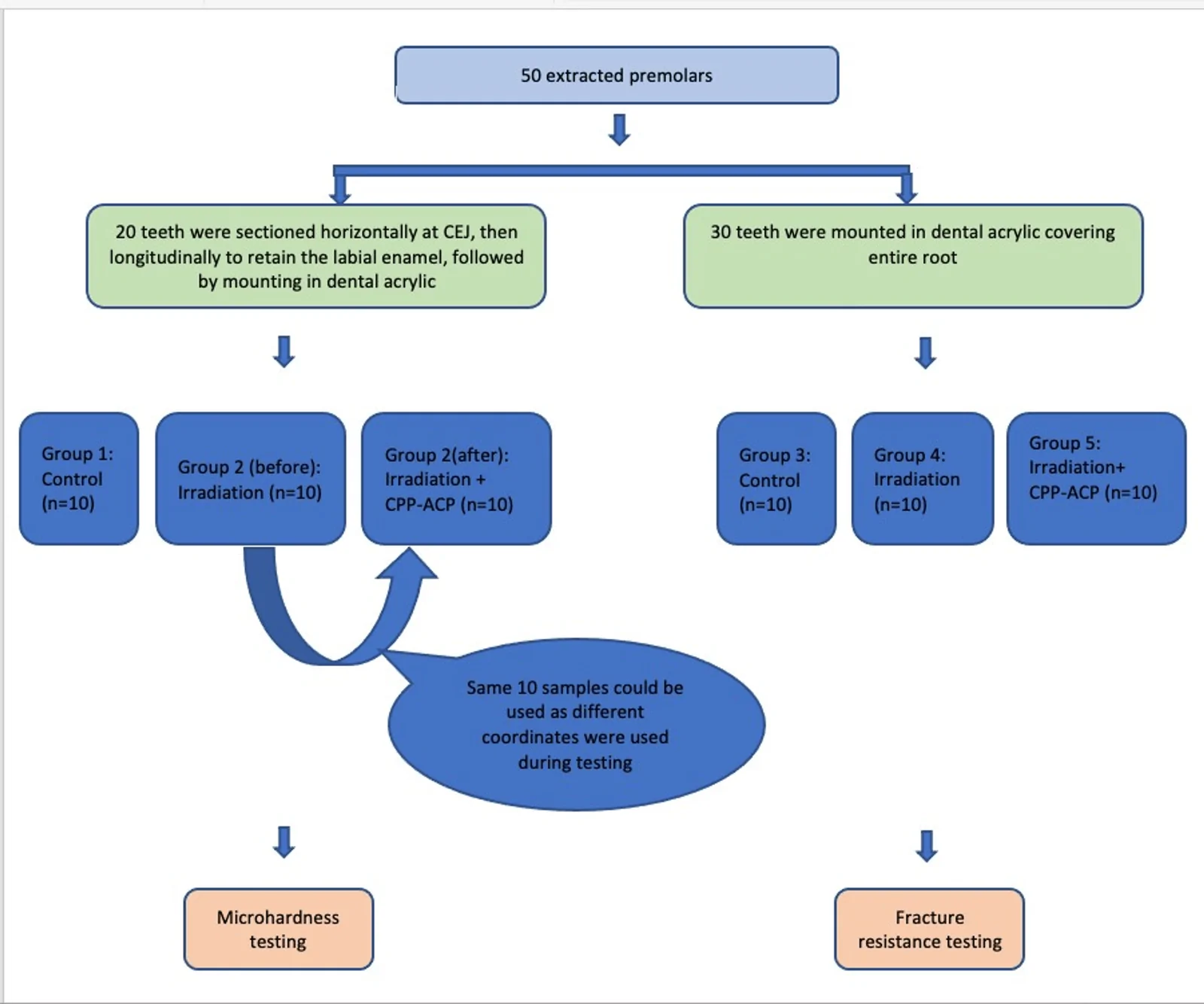
Study flowchart. CEJ: cementoenamel junction; CPP-ACP: casein phosphopeptide-amorphous calcium phosphate

Fifty extracted human premolar teeth were collected from the Department of Dentistry of People's College of Dental Sciences and Research Centre in Bhopal, India. Ethical clearance for human-derived products was obtained from the Institutional Ethical Committee of People's College of Dental Sciences and Research Centre (approval number: PCDS/IEC/2021/1/602) prior to commencement of the study. The teeth were extracted for orthodontic reasons and were intact. None of the teeth had caries, cracks, defects, resorption, attrition, or restoration. All the selected teeth were debrided and cleansed using an ultrasonic scaler (Woodpecker, Guilin Woodpecker Medical Instrument Co., Ltd., Guilin, China) to remove the soft tissues and calculus flecks and polished with a low-speed handpiece (NSK, Nakanishi Inc., Kanuma, Japan) and rubber cup (Cotisen, Huanghua Promisee Dental, Huanghua City, China). All the samples were kept in saline solution (Aculife, Aculife Healthcare, Ahmedabad, India) at 37°C and 95% humidity to simulate physiological conditions until use. In order to test for fracture resistance, 30 teeth were embedded in dental acrylic (DPI, New Delhi, India) covering the entire root, leaving the crown exposed at the level of the cementoenamel junction (CEJ). For microhardness testing, 20 teeth were prepared by sectioning at the CEJ using a diamond disk (Mani, Inc., Tochigi, Japan) with water. The crowns were sectioned longitudinally so as to keep the labial enamel, followed by mounting in dental acrylic (Figure 1).

Irradiation procedure

**Figure 2:**
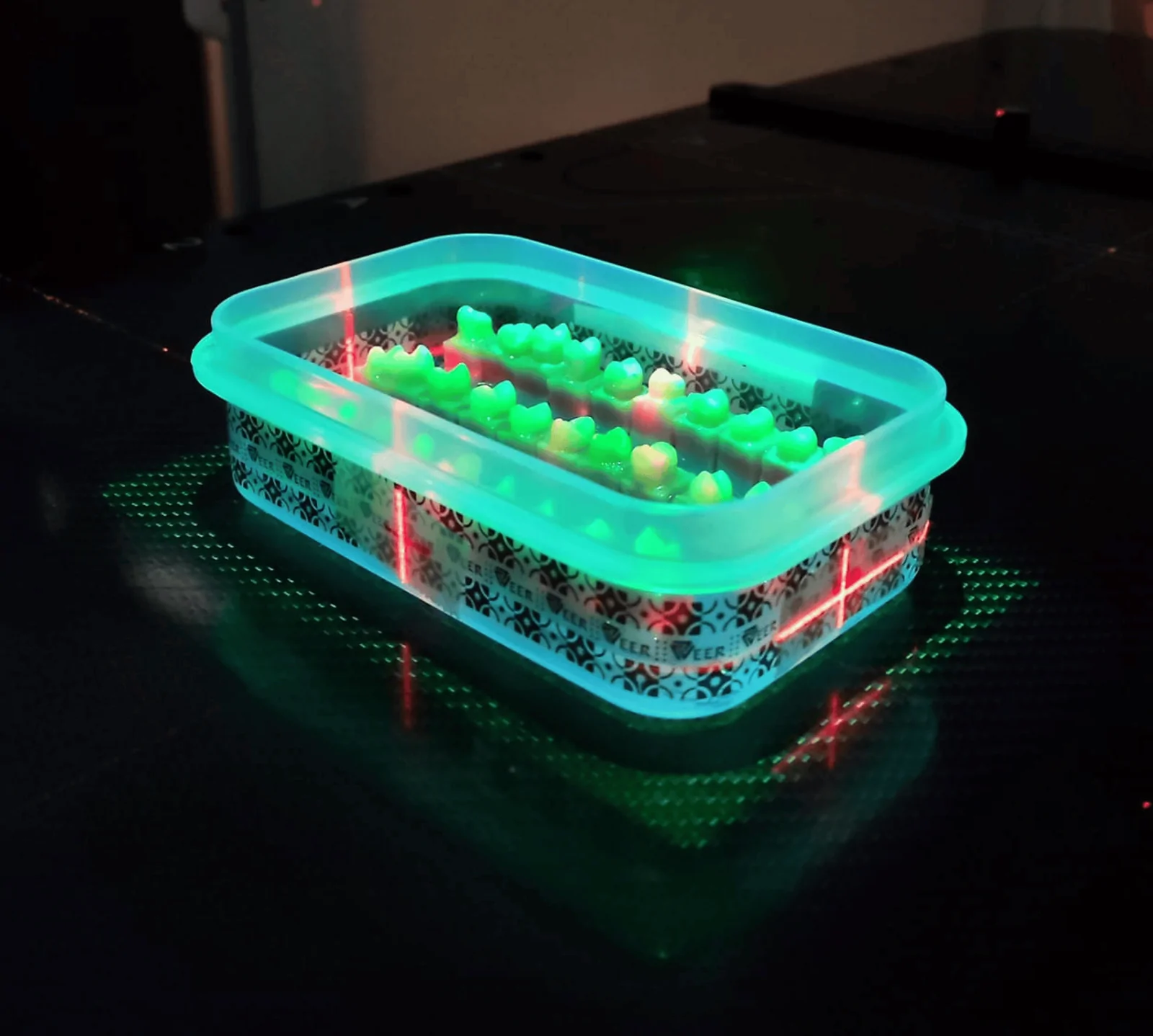
Irradiation of samples using a linear accelerator (Elekta Infinity, Elekta AB, Stockholm, Sweden).

The radiotherapy protocols for head and neck cancer patients correspond to 70 Gy. In this study, the samples were irradiated for 35 fractions with 2 Gy exposure per day applied five days per week, for seven weeks with X-rays from a linear accelerator (Elekta Infinity, Elekta AB, Stockholm, Sweden) in the Department of Radiotherapy (Figure 2).

**Figure 3:**
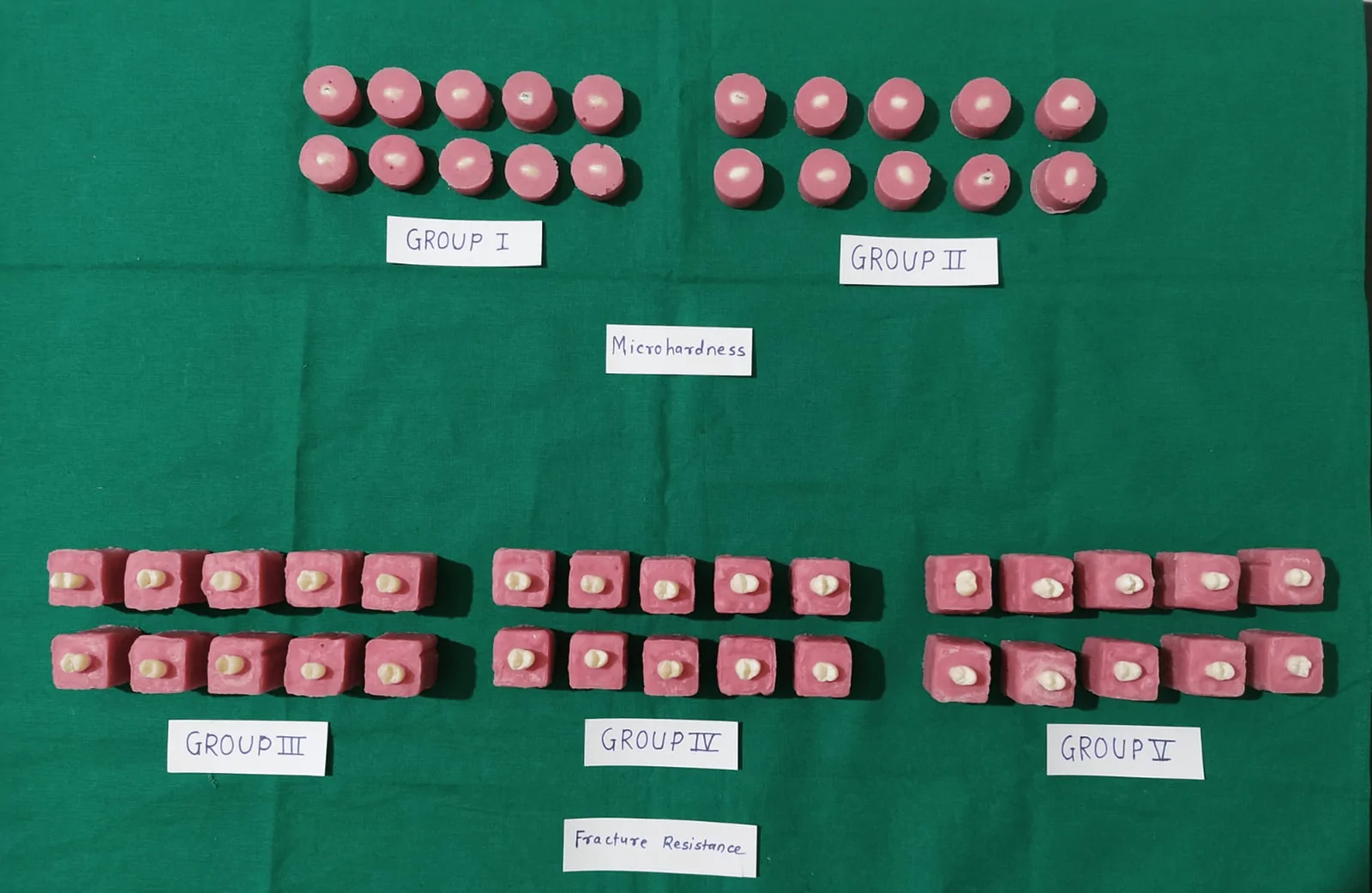
Five groups of samples (n=10).

The teeth were randomly divided into five groups (n=10) (Figure 3).

All the teeth were submerged in artificial saliva (Arosa-Duo, Arosa Biotech, Thane, India). In group 1, the samples were not irradiated and were subjected to microhardness testing. In group 2 (before), the samples were irradiated, followed by microhardness testing. In group 2 (after), CPP-ACP (GC Tooth Mousse, GC Corporation, Tokyo, Japan) was applied onto the tooth surface using a microbrush and left for three minutes and rinsed with saline afterward. The teeth were subjected to microhardness testing. In group 3, the samples were not irradiated and the agent was not applied. The samples were subjected to fracture resistance testing. In group 4, the samples were irradiated, followed by fracture resistance testing. In group 5, the samples were irradiated. CPP-ACP was applied to the enamel surface using a microbrush and left for three minutes and rinsed with saline afterward. The samples were subjected to fracture resistance testing.

Different coordinates were used in group 2 before and group 2 after sample during testing, making sure that the same area was not indented again. Because of this variation, the same 10 samples could be used before and after medicament application.

Surface microhardness testing

**Figure 4:**
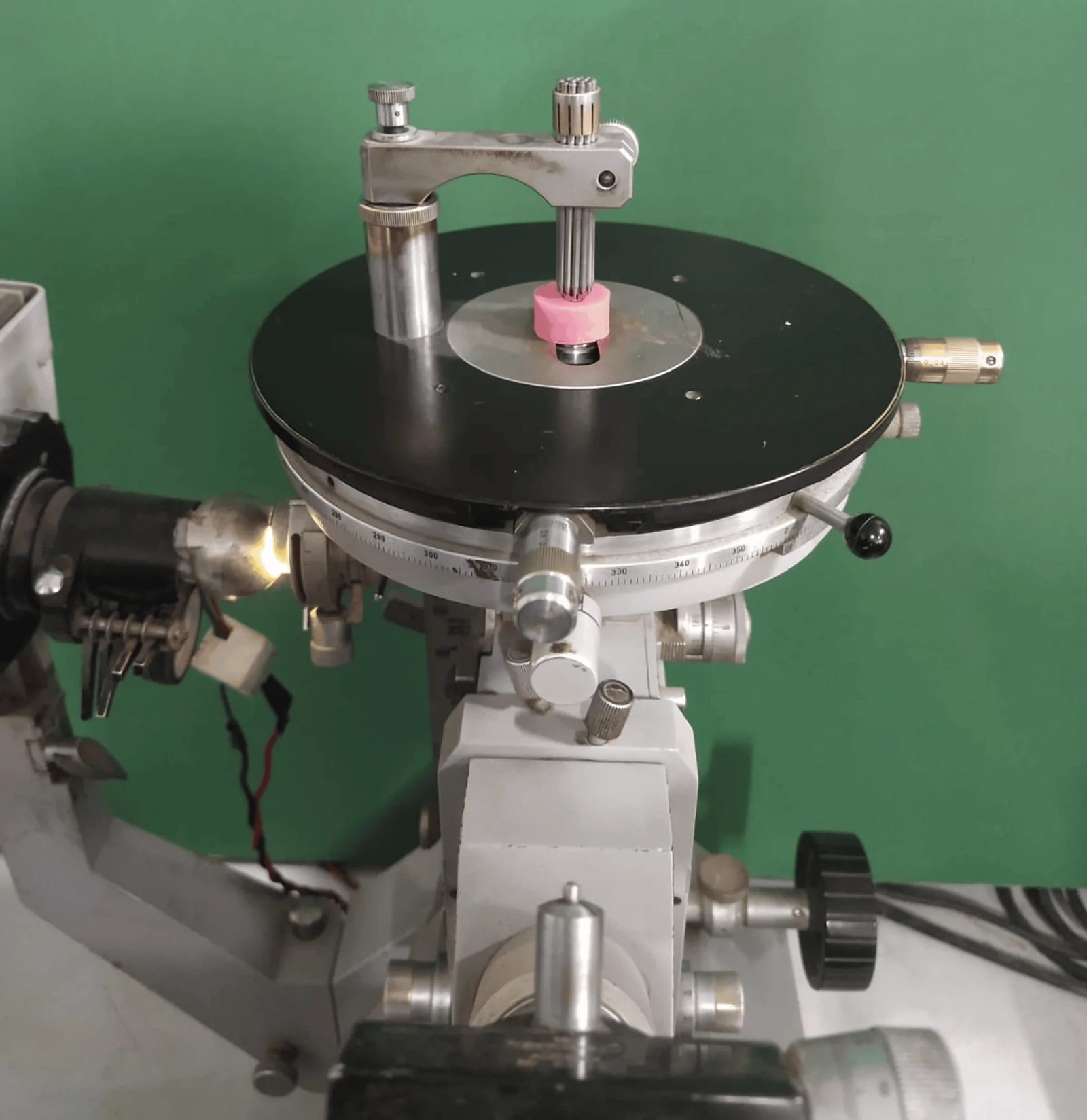
Microhardness testing of sample using a microhardness tester (Reichert Austria Make, Reichert, Vienna, Austria).

The surface microhardness of the tooth was tested using a microhardness tester (Reichert Austria Make, Reichert, Vienna, Austria; Sr. No. 363798; reference standard: ISO 6507) with a load of 100 g (Figure 4).

Fracture resistance testing

**Figure 5:**
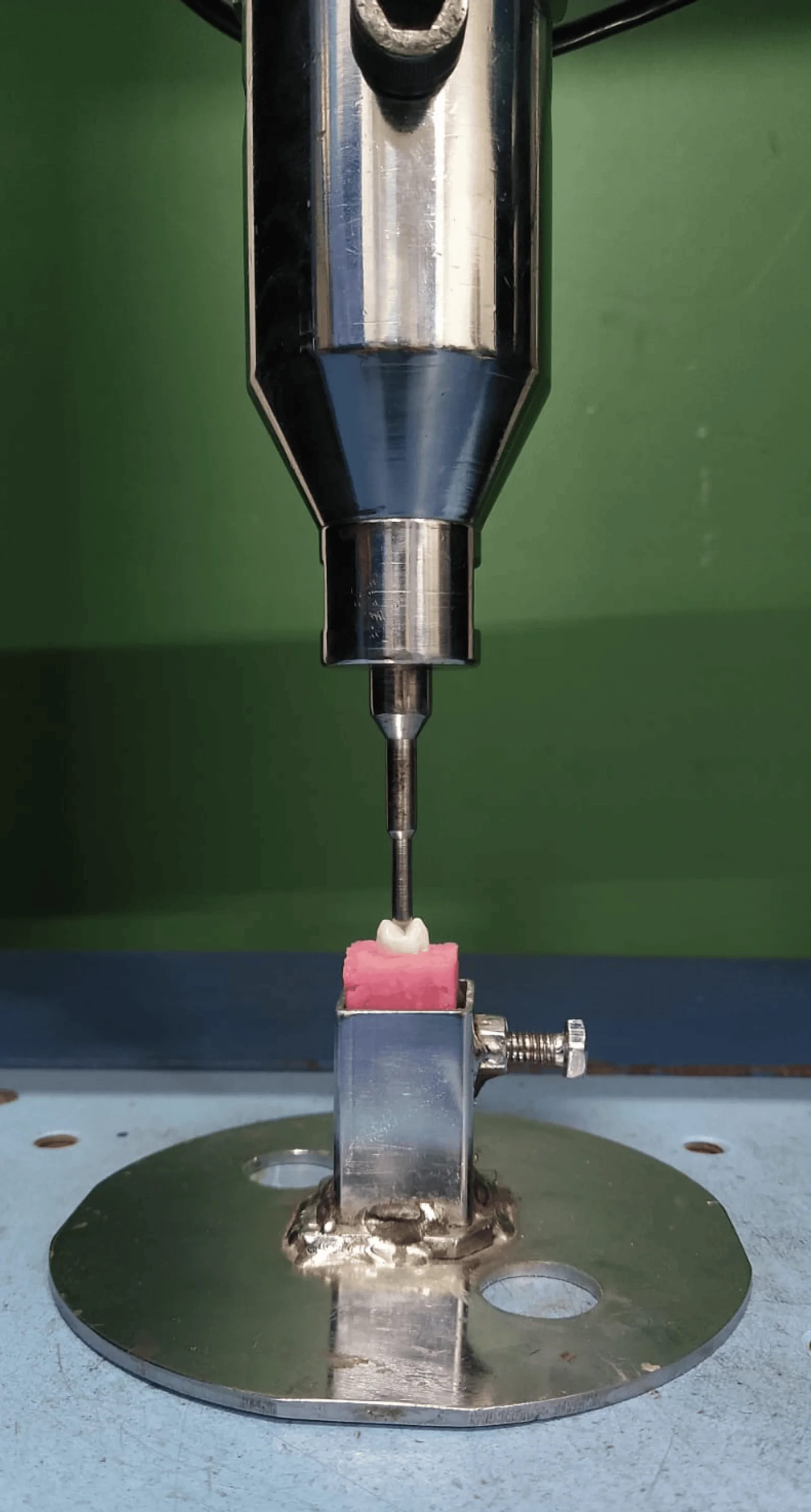
Fracture resistance testing of sample using a universal testing machine (ACME Engineers, New Delhi, India; model: UNITEST 10).

The fracture resistance of the tooth was measured using a universal testing machine (computerized, software-based; ACME Engineers, New Delhi, India; model: UNITEST 10; system accuracy of the machine: ±1%) with a crosshead speed of 0.5 mm/minute and a plunger diameter of 3 mm (Figure 5).

Data analysis

The data analysis was done using IBM SPSS Statistics for Windows, V. 26.0 (IBM Corp., Armonk, NY, USA). The recorded values were statistically analyzed using the Kruskal-Wallis test and Mann-Whitney U test. The level of significance was set at 5% (p-value).

## Results

The study included 50 samples. These samples were divided into five groups, with each group including 10 samples. The first two groups of samples were used to measure the microhardness, and the other three groups were used to measure the fracture resistance. During the testing of microhardness for group 2 before and group 2 after sample, different coordinates were used, ensuring that the same area was not indented again. Because of this variation, the same 10 samples could be used before and after medicament application. Hence, 50 samples served the purpose of 60 samples leading to the formation of six groups during testing.

Microhardness analysis

The mean microhardness of the control group was 323.50±7.76, the irradiation group was 269.60±9.41, and the irradiation and medicament group was 288.90±10.10. Kruskal-Wallis test showed a significant difference between the groups with a chi-squared value of 24.24 and a p-value of 0.001 (Table 1 and Table 2). Mann-Whitney U test showed a significant difference between each pair of groups. Therefore, the microhardness was maximum in the control group. Irradiation significantly decreases the microhardness, but application of medicament improves the microhardness, but not to the level of the natural tooth.

**Table 1:** Comparison of microhardness and fracture resistance between the groups. **Highly significant, Kruskal-Wallis test

Properties	Group	N	Mean	SD	Chi-squared value	P-value
Microhardness (HV)	Control	10	323.50	7.76	24.24	0.001**
	Irradiation	10	269.60	9.41		
	Irradiation and medicament	10	288.90	10.10		
Fracture resistance (N)	Control	10	1315.35	163.43	23.81	0.001**
	Irradiation	10	835.10	75.46		
	Irradiation and medicament	10	1010.35	76.69		

**Table 2:** Comparison of microhardness and fracture resistance between each pair of groups. **Highly significant, Kruskal-Wallis test

Comparing groups		Microhardness	Fracture resistance
				Control	Irradiation	0.001**	0.001**
Control	Irradiation and medicament	0.001**	0.001**
Irradiation	Irradiation and medicament	0.001**	0.001**

Fracture resistance analysis

The mean fracture resistance of the control group was 1315.35±163.43, the irradiation group was 835.10±75.46, and the irradiation and medicament group was 1010.35±76.69. Kruskal-Wallis test showed a significant difference between the groups with a chi-squared value of 23.81 and a p-value of 0.001 (Table 1 and Table 2). Mann-Whitney U test showed a significant difference between each pair of groups. Therefore, the fracture resistance was maximum in the control group. Irradiation significantly decreases the fracture resistance, but application of medicament improves the fracture resistance, but not to the level of the natural tooth.

## Discussion

This study showed that radiation has a detrimental effect on the mechanical properties of enamel and an anti-cariogenic agent plays an important role in the prevention of radiation-induced caries. The purpose of this study was to assess whether an anti-carious agent has any remineralizing effect on teeth or not. The testing involved changes in mechanical properties before and after irradiation and after medicament application, at a fixed radiation dose of 70 Gy. We did not consider the dosage aspect of the remineralizing agent as well as radiation. The null hypothesis was rejected.

The results of this study revealed that the microhardness significantly (p<0.05) decreases after irradiation. As opposed to irradiated teeth, the microhardness of medicament-treated teeth was significantly higher; however, it was not as high as that of natural teeth. Some studies have shown changes in the microstructure of dentin leading to cracks and fractures. The peritubular and intratubular dentin collapse, causing the occlusion of the dentinal tubules [10,11]. There is an increase in the stiffness of dentin and enamel near the dentinoenamel junction due to radiation-induced reduction in protein content. This change is more prominent in enamel sites, resulting in wearing off near the cervical area, making it more susceptible to caries. Fränzel et al. conducted a study to assess the effect of irradiation and induced xerostomia on enamel and dentin. They kept the teeth in acidic hydroxyl cellulose gel to induce demineralization in order to simulate xerostomic condition and kept the radiation exposure at 60 Gy. They found that the effect of demineralization is minuscule on irradiated teeth as compared to non-irradiated teeth. The hardness of irradiated enamel is 6%, and the elastic modulus is 39% of the non-irradiated teeth values. The mechanical properties are severely affected by radiation; however, xerostomia plays a secondary role only [12]. Various factors like hyposalivation, poor oral hygiene, cariogenic diet, and direct effect of radiation on dental hard tissue play a role in the manifestation of radiation-induced caries [13].

Seyedmahmoud et al. conducted a study using in vitro and in vivo irradiation on three levels of enamel: outer, middle, and inner layers. They found that radiation decreases the microhardness of all sites in enamel; however, this reduction is significantly (p<0.05) present in outer and middle enamel [14]. Hence, outer enamel was the region of interest in this study. Relation of dose and mechanism of damage is controversial in different studies. According to Muñoz et al., radiation affects the inorganic portion of dentin and enamel in the same manner. The ratio between carbonate and phosphate alters, causing changes in the hydroxyapatite crystal at doses of 40 Gy and above. Changes in the organic matrix of enamel start at doses as low as 20 Gy [15]. However, in another study by Markitziu et al., radiation doesn't affect the inorganic part of enamel; rather, it damages the organic part by free radical formation. Changes in enamel are a result of damage to the interprismatic portion [16].

According to Lu et al., a combination of premature damage to enamel adjacent to the dentinoenamel junction and reduced crystallinity of enamel are responsible for radiation-induced caries [11]. Considering the studies mentioned above, it is not certain whether radiation affects the organic or the inorganic portion of enamel. However, it can be understood that radiation does affect the tooth structure in such a manner that the integrity is lost, making it susceptible to caries. Fracture resistance is a mechanical property that describes the resistance of brittle materials to the propagation of cracks under a load [17]. Increased stiffness makes teeth brittle; hence, fracture resistance is an important mechanical parameter for irradiated teeth. There are several studies done on the mechanical and structural changes in dentin, but less on enamel [18]. Enamel microhardness can be measured by either Vickers hardness number (VHN) or Knoop hardness number (KHN). VHN doesn't depend on the load; however, KHN does [19]. Therefore, it was decided to calculate VHN and convert it to Vickers pyramid number (HV number). VHN is a measure of resistance to indentation.

It is of paramount importance to prevent caries at its earliest in cancer patients prior to radiotherapy. Many factors like sudden multiple teeth involvement, reduced patient compliance, and complicated medical history make treatment more challenging. Also, if restoration is done after radiotherapy cycles, there are chances of failure due to collagen damage. Ionizing radiation on the water in tissues produces free radicals. The microtensile bond strength between the tooth and composite tends to decrease because free radicals inhibit the polymerization of adhesive agents, leading to adhesive failure [20,21].

Fluoride has been the gold standard for anti-cariogenic agents for decades. It improves remineralization and reduces demineralization. It is available as varnishes, mouth rinses, dentifrices, and gels [22]. However, in the last few years, a distinguished product based on nanoscience has been available, CPP-ACP. Fluoride use may present with fluorosis if used excessively. However, CCP-ACP is a safer choice with fewer chances of side effects [23]. Clinicians must be vigilant with patients having milk allergies as CPP-ACP is a milk product. According to Mazzaoui et al., CPP-ACP when combined with fluoride has a synergistic action [24]. Yamaguchi et al. conducted an in vitro study and concluded that CPP-ACP enhances remineralization and decreases demineralization of enamel [25]. Oshiro et al. performed a study on bovine teeth and found that teeth treated with CPP-ACP showed less demineralization [26]. However, the amount of time required for achieving the remineralization process using this agent is not clear.

Lieshout and Bots found that apart from hyposalivation, the direct effect of radiation is also responsible for caries formation. So, even if the exact clinical scenario of hyposalivation cannot be simulated, it is important to study the effect of radiotherapy on the mechanical properties of teeth [27]. Artificial saliva is usually carboxymethyl cellulose- or mucin-based, which has a wetting ability. The one used in this study consists of sodium carboxymethyl cellulose, sorbitol, and various ionic substances like potassium chloride, sodium chloride, etc. [28]. Water is considered as a reference medium for absorption dose measurement in radiotherapy. However, water-equivalent plastics like polymethmethacrylate, plastic, and polystyrene can also be used. This is why we used acrylic for mounting the teeth and a plastic box for radiation purposes [29].

This study has several limitations. Firstly, it is designed around an in vitro model, hence lacking the authenticity of an in vivo study. Secondly, there is an inability to simulate a xerostomic condition. Along with a decrease in the quantity of saliva, other factors like reduced flow rate, a shift towards acidogenic microflora, and altered composition also contribute to the formation of radiation-induced caries [3]. Thirdly, the effect of a cariogenic diet on the development of caries was not assessed. Fourthly, we did not consider the dosage aspect of radiation exposure. Further studies are required to circumvent these deficiencies.

## Conclusions

According to the findings of this study, we conclude that a radiation dose of 70 Gy alters the microhardness and fracture resistance of enamel. The application of remineralizing agent CPP-ACP significantly improves the microhardness and fracture resistance of irradiated enamel. However, the application of CPP-ACP doesn't improve the microhardness and fracture resistance up to the level of a natural tooth.
